# A historical review of the techniques of recovery of parasites for their detection in human stools

**DOI:** 10.1590/0037-8682-0535-2019

**Published:** 2020-06-01

**Authors:** Felipe Augusto Soares, Aline do Nascimento Benitez, Bianca Martins dos Santos, Saulo Hudson Nery Loiola, Stefany Laryssa Rosa, Walter Bertequini Nagata, Sandra Valéria Inácio, Celso Tetsuo Nagase Suzuki, Katia Denise Saraiva Bresciani, Alexandre Xavier Falcão, Jancarlo Ferreira Gomes

**Affiliations:** 1 Universidade Estadual de Campinas, Faculdade de Ciências Médicas, Campinas, SP, Brasil.; 2 Universidade Estadual Paulista, Faculdade de Medicina Veterinária, Departamento de Apoio, Produção e Saúde Animal, Araçatuba, SP, Brasil.; 3 Immunocamp Ciência e Tecnologia, Campinas, SP, Brasil.; 4 Universidade Estadual de Campinas, Instituto de Computação, Campinas, SP, Brasil.

**Keywords:** Parasitology, Intestinal diseases, Historical aspects, Routine diagnostic test

## Abstract

Since the early 20^th^ century, the detection of intestinal parasites has improved with the development of several techniques for parasitic structures recovery and identification, which differ in sensitivity, specificity, practicality, cost, and infrastructure demand. This study aims to review, in chronological order, the stool examination techniques and discuss their advantages, limitations, and perspectives, and to provide professionals and specialists in this field with data that lays a foundation for critical analysis on the use of such procedures. The concentration procedures that constitute the main techniques applied in routine research and in parasitological kits are a) spontaneous sedimentation; b) centrifugation-sedimentation with formalin-ethyl acetate; and c) flotation with zinc sulfate solution. While selecting a technique, one should consider the purpose of its application and the technical-operational, biological, and physicochemical factors inherent in the procedures used in stool processing, which may restrict its use. These intrinsic limitations may have undergone procedural changes driven by scientific and technological development and by development of alternative methods, which now contribute to the improvement of diagnostic accuracy.

## INTRODUCTION

Human intestinal parasites are a global public health issue, primarily when associated with poor sanitary and socioeconomic conditions in tropical and subtropical climate areas^1^ ^,^ ^2^. Diseases caused by these parasites are endemic in less developed communities in various peripheral regions of the planet^3^. In 2010, the World Health Organization (WHO) reported these infectious diseases as part of the portfolio of major neglected tropical diseases worldwide^4^. Factors related to increased tourism and global migration, zoonotic diseases, basic sanitation, water supply, hygienic habits, local culture, immunological status of the population, and life cycle and adaptation of the parasites to their hosts in adverse environmental conditions determine the maintenance of these infections in populations^5^ ^-^ ^7^.

Currently, around 18 human-pathogenic species have a cosmopolitan distribution because of exposure to socioenvironmental risk factors^8^ ^-^ ^10^. Intestinal parasitic infections require constant monitoring, control, and treatment as they impair the ability of people to work, leading to economic loss and a high burden to the public health services^4^ ^,^ ^5^ ^,^ ^11^.

The etiological agents causing these human parasitic diseases are classified into two broad groups: protozoa (amebae, flagellates, ciliates, and coccidia) and helminths (nematoda, cestoda, and trematoda)^8^. *Entamoeba histolytica*/*E. dispar*, *Giardia* spp*.*, and *Cryptosporidium* spp. are among the most commonly reported parasites in pre-school and school-age children in developing countries with poor sanitation and hygiene conditions^12^ ^,^ ^13^. These protozoa cause high morbidity in immunocompromised patients^14^. The genus *Cryptosporidium* alone was associated with an annual mortality of about 520,000 children worldwide, in addition to intermittent diarrhea and malnutrition^15^ ^-^ ^17^. The soil-transmitted helminths, including *Ascaris lumbricoides, Trichuris trichiura, Ancylostoma duodenale,* and *Necator americanus*, occur in single or mixed infections, with at least two species of parasites present in the same individual. These are found in more than one billion people in sub-Saharan Africa, Latin America, China, and East Asia^18^.

Diseases due to parasitic infections are related to the degree of virulence of the species^3^, to the intensity of the infection^19^, and to the quality of individual immune response^20^. Symptoms may range from hyporexia, anorexia, diarrhea, intestinal obstruction, and rectal prolapse to anemia and reduced micronutrient absorption^3^ ^,^ ^21^. Due to non-specific manifestations, the definitive diagnosis in laboratories^22^ is necessary, either by identifying cysts, oocysts, and spores of protozoa; eggs and larvae of helminths; or by detecting parasite DNA or RNA in patients’ biological samples^8^ ^,^ ^23^.

Among the available laboratory analyses, stool examination has a lower operational cost, with moderate sensitivity and specificity^24^, and is therefore recommended by the WHO for government control programs and epidemiological surveys^17^ ^,^ ^25^ ^,^ ^26^. At low infection intensities, when few parasites are excreted in stools, this examination accuracy can be increased with technical procedures that eliminate most of the fecal debris^23^ ^,^ ^27^. 

Thus, this work aims to review the main historical moments and perspectives of different technical procedures for parasitological diagnosis by microscopy in stool samples according to their advantages and limitations. 

## HISTORY OF TECHNICAL PROCEDURES OF PARASITE RECOVERY IN STOOL SAMPLES

 Since 1908, several qualitative techniques have been developed to improve parasitological stool diagnosis. These were based on separating solids and liquids in fecal suspension to eliminate excess debris and obtain abundant sediment containing parasites. Therefore the gravity force and specific density of a liquid medium taken into account^28^. Other technical devices developed after hundred years of research allow quantitative detection of eggs of up to five species of intestinal helminths in human feces and determine the infection intensity in an individual^29^. Thus, since the early 20^th^ century, scientists have been searching for methods to improve stool examination with higher sensitivity and indexes closer to reality^27^ ^-^ ^29^.

There are five technical principles that underly clinical analysis: centrifugal sedimentation^30^; centrifugal flotation^31^; spontaneous flotation^32^ ^,^ ^33^; spontaneous sedimentation^34^ ^,^ ^35^; and positive thermo-hydrotropism of intestinal nematode larvae^36^ ^-^ ^38^. Due to high daily demand for stool examinations in clinical laboratories, the use of commercial kits has become prevalent as it is a practical and low-cost option^23^ ^,^ ^27^.

Qualitative and quantitative laboratory technical procedures were consolidated when majority of the population in developing countries were denied access to basic sanitation, treated water, sanitary education, and adequate drug intervention. All of those conditions influenced the high intensities of intestinal parasite infections^5^ ^,^ ^11^ ^,^ ^39^. However, with time, these infections and their intensities have begun to decrease due to the improvement of living conditions, increasing urbanization, and increasing drug treatment for the population. Consequently, the conventional diagnostic techniques have lost sensitivity^4^ ^,^ ^40^. 

Several integrated technical parameters can increase the diagnostic sensitivity during low concentrations of parasitic structures in fecal suspension, such as force g (spontaneous or with centrifugal action); time of application in sedimentation and/or flotation procedure; specific density of the medium; measurement of filter-mesh orifice; chemical characteristics of the preservative solution; type of coloring (temporary and permanent); and portion of the collected fecal sample^27^ ^,^ ^41^ ^,^ ^42^. These parameters need to be carefully evaluated for the precise laboratory validation of different diagnostic techniques and/or kits^24^ ^,^ ^26^ ^,^ ^27^ ^,^ ^41^ ^,^ ^43^. Similarly, it was verified that the use of the term "concentration" that characterizes some of the well-known parasitological techniques in the literature needs to be considered carefully since the studies of comparison and validation of these techniques do not determine the amount or concentration of parasites found in the aliquot of the fecal suspension evaluated^27^ ^,^ ^30^ ^,^ ^31^ ^,^ ^35^.

## TECHNICAL PRINCIPLES FOR STOOL PARASITOLOGICAL DIAGNOSIS

The parasitological techniques employed effectively in clinical laboratory routines allow identification of cysts, oocysts, helminth eggs, and nematode larvae from fecal samples^8^ ^,^ ^22^ ^,^ ^44^. However, the technical principle should be selected considering its purpose (research, teaching or laboratory routine), sensitivity, specificity, speed of execution and practicality, equipment operational cost, infrastructure, personnel training, technical capacity, experience for the analytical demands and analyses of results^23^ ^,^ ^45^ ^,^ ^46^. 

Even if studies define a technique as a “gold standard”^17^, in general, it is advisable to compare the results obtained by two or more sensitive techniques as a “gold standard” diagnostic result^24^ ^,^ ^26^ ^,^ ^47^ ^,^ ^48^. When we consider the specific aspects of the methods mentioned above, stool parasitological diagnosis still continues without a “100% effective” research tool^24^ ^,^ ^47^.

Biotechnology techniques are essential tools in certain situations^49^ ^,^ ^50^; however, the use of molecular or immunological methods to detect parasites in fecal samples may not meet the economic and operational requirements of a laboratory, especially in regions with limited resources^17^ ^,^ ^25^ ^,^ ^26^ ^,^ ^50^ ^-^ ^52^. Large reference centers can adopt these more sophisticated methods for research and differentiation between morphologically similar species^49^ ^,^ ^51^.

Stool examination by using conventional techniques or commercial kits^41^ ^,^ ^45^ ^,^ ^53^ ^,^ ^54^, which may have low sensitivity, are characterized by ease of performance and low cost for direct identification of infective agents^43^ ^,^ ^55^ ^,^ ^56^, and remain the most performed, widespread, and appropriate methods to detect human intestinal parasites^23^ ^,^ ^50^ ^,^ ^57^. However, *Microsporidium* spp. (1-2.5 µm), *Cryptosporidium* spp. (4-6 µm), *Cyclospora* spp. (8-10 µm), and *Cystoisospora* spp. (20-30 µm) are difficult to detect by the conventional parasitological techniques. A major obstacle in the detection of these parasites is the need to perform specific permanent staining techniques^23^ that require specialized personnel and are time-consuming and labor intensive^58^. In addition, errors in test interpretation can occur because the parasitic structures (oocysts) are much smaller than larvae and helminth eggs. Therefore, clinicians may easily mistake real oocysts for yeasts or debris^23^, except for *Cystoisospora* spp.

Technological development and combining alternative methods have improved the diagnostic accuracy of these tests. For instance, the coproparasitological examination begins with sample collection and follows specific technical recommendations to obtain a sample with the best quality, regardless of the type of method used. Storing specimens for transportation to the analysis site may also interfere with the quality of examination and result^47^ ^,^ ^59^ ^,^ ^60^. The sample quality is verified by detecting conserved structures of the agents in the sample using 5% to 10% buffered formalin-based solutions, polyvinyl alcohol, and merthiolate-iodine-formalin as described by Sapero and Lawless^61^, and modified by Blagg et al.^62^ with sodium acetate-acetic acid-formalin^23^ ^,^ ^63^. The 3:1 ratio of preservative to fecal material preserves the sample from deterioration^8^ ^,^ ^47^; it is recommended to constantly refrigerate unpreserved material between 3°C and 5°C in an air-tight container^47^ ^,^ ^59^. 

### Centrifugation-sedimentation

The centrifugal-sedimentation principle used in the laboratory for the recovery of intestinal parasites from fecal samples was first reported in 1908 by Telemann, when centrifugal force was applied to a suspension containing ether (solvent), hydrochloric acid, and fecal material to separate the specific portion of the feces containing intestinal parasites^64^. Thenceforth, the procedures and technical parameters have been modified, with addition or simplification of processes^27^.

In the 1940s, Lawrence Ritchie presented an alternative strategy for fecal sample preparation to that reported by Telemann. In this procedure, preservative-free feces were diluted in saline solution and filtered through two layers of gauze, followed by repeated washes by centrifugation and resuspension of the sediment that, in the last stage of separation, was preserved in 10% formalin with ethyl ether addition^30^. Thus, layers of ether, light fecal debris, formalin, and sediment containing organic material and parasitic structures are obtained^8^. Through this procedure, known as the formalin-ether concentration technique, the parasitic forms are best conserved by avoiding acidic reagents that could affect their morphology^30^, enabling a cleaner observation field under microscopy^27^.

In 1953, Maldonado and Acosta-Matienzo added neutral detergent to the procedure described by Ritchie in 1948. This surfactant effect helped emulsify the fat molecules of the sample^65^. This enabled the sediment to become less dense than organic matter and suppressed the adherence of the parasites to the debris, thus allowing easier identification of the parasitic structures^27^ ^,^ ^66^. 

In 1970, Allen and Ridley suggested further modifications to simplify what was suggested by Ritchie by centrifuging the sample at 3000 rpm for one minute with improved filtration^67^ due to the risk of flammability, volatility, explosion, and environmental toxicity by ethyl ether. Young et al. recommended replacing this product with ethyl acetate, which improved the recovery of parasite structures in comparison with Ritchie’s method, since it did not alter the morphology of cysts, eggs, and parasite larvae^68^. This new procedure of centrifugal-sedimentation of fecal samples for intestinal parasite research was then reported as formalin-ethyl acetate sedimentation concentration technique^23^.

The performance of the centrifugal-sedimentation processes compared with other techniques/methods was reported by Yanet et al. They described the formalin-ether concentration technique with a sensitivity of 100 and 68.13% when compared with that of the direct method and spontaneous sedimentation using the Paratest® kit, respectively^69^. According to Speich et al., the formalin-ether concentration technique showed similar sensitivity, and in some cases, it was superior to that presented by the Kato-Katz technique with a single sample for geohelminths detection^70^. Hussein et al. reported the formalin-ether concentration technique showing 98.2% accuracy compared with 67.5% accuracy obtained by Kato-Katz technique^71^. A similar result was demonstrated by Hailu & Abera, who reported a 92.3% sensitivity with formalin-ether concentration, 61.1% sensitivity with direct smear, and 58.7% sensitivity with Kato-Katz technique^72^. 

The sensitivity of a technique may be influenced by the use of several reagents during the procedure that enable the ionic charge of the fecal suspension to favor adhesion of a part of the parasites that is to be retained in the upper layers of the sediment^27^ ^,^ ^41^ ^,^ ^46^. For this reason, some aspects of the procedure must be overcome in the formalin-ether concentration technique, such as loss of parasites in the liquid medium and morphological alterations of parasitic structures during the process, although the technique has undergone modifications since its creation.

### Spontaneous flotation and centrifugal flotation

The spontaneous flotation technique was developed by Bass to detect hookworm eggs^73^. The principle of the technique is based on the difference between the density of a specific flotation solution and that of parasitic structures such as eggs, helminth larvae, and protozoan cysts and oocysts. Several tests with different solutes resulted in a variety of density parameters that encompassed the structures of the several species of intestinal parasites until research established consensual values between 1.036 g/mL and 1.225 g/mL^74^ ^-^ ^77^. The spontaneous flotation technique was then performed with saturated solutions with a density between 1.18 g/mL and 1.27 g/mL, depending on the reagent used^8^. Recovery of light helminth eggs in supernatants of fecal samples suspended in sodium chloride solution (NaCl, 1.20 g/mL) has also been reported by Willis^32^. However, crystal formation in the fecal smear and plasmolysis of protozoa by osmotic pressure when exposed to the saturated solution of salt makes this technique inappropriate for the recovery of dense eggs and protozoa^59^ ^,^ ^66^ ^,^ ^78^.

In 1923, Leslie Sheather tested concentrated sucrose solution (1.20 g/mL) instead of sodium chloride solution and improved Willis’ technique by centrifuging the mixture and recovering coccidian protozoa of the genus *Isospora* and cysts of *Balantidium* spp.^33^. However, this adaptation made the sucrose solution viscous and decreased the recovery of some parasitic forms^66^ ^,^ ^79^. Then, in 1939, Faust et al*.* performed centrifugal flotation with concentrated zinc sulfate solution (1.18 g/mL) and recovered twice as many cysts when compared to that by direct examination, and 80% of the helminths in an evaluation between 10 different coproparasitological techniques^31^. From this result, this technique was chosen as a reference for worldwide investigation and intestinal parasites surveys.

The use of flotation techniques to recover light helminth eggs is effective and more frequent that sedimentation/centrifugation for the detection of helminths in livestock and commercial animals^42^ ^,^ ^46^; however, these techniques are restricted to nematode species (*Toxocara* spp*., Ancylostoma* spp*., Trichuris* spp., for example) and protozoa detection^46^ ^,^ ^48^. The frequent reports of distortion in protozoa such as *Giardia duodenalis* and *Entamoeba* spp., and the disruption of eggshells, such as those of *Schistosoma mansoni* and *Hymenolepis* spp*.* have demonstrated that using saturated solutions of reagents for flotation technique is not recommended for the laboratory detection of intestinal parasites in animals^59^ ^,^ ^66^ ^,^ ^68^ ^,^ ^80^ ^-^ ^85^.

### Spontaneous sedimentation

The spontaneous sedimentation technique uses the separation between solid and liquid media of different densities for the detection of infectious agents in feces and urine. Detection of intestinal parasites is done by preparing a homogeneous mixture containing five grams of feces diluted in distilled water, followed by filtration of the fecal suspension and a 12-hour sedimentation^24^. The supernatant liquid is then decanted until the sediment containing the parasites is isolated^35^ ^,^ ^47^.

The technique was mentioned by Adolfo Lutz, in 1919, when reporting its usefulness in detecting eggs of *S. mansoni* as part of his study, which is of recognized relevance for parasitology since it elucidated the clinical and epidemiological aspects of schistosomiasis in Brazilian and African territories^34^. Furthermore, for *S. mansoni* detection, Hoffman, Pons, and Janer observed a 20% superiority in sedimentation compared to direct examination^35^. In this publication, they provided a detailed method for spontaneous fecal material sedimentation, the economic and operational feasibility of which is well known^86^. However, the spontaneous sedimentation technique showed 33%, 49%, and 68% sensitivity in studies with intestinal parasites^24^ ^,^ ^43^ ^,^ ^69^ and is considered low to moderate when compared to other techniques, especially in cases of reduced parasite load. Sedimentation can be applied on a large scale by using parasitological kits (for example, Paratest^®^ and Coprotest^®^), and contamination of samples can be prevented by using disposable components and handling samples in a closed system^55^ ^,^ ^56^.

In this technique, sedimentation time may be the limiting factor for its use in large-scale laboratory routines. Another disadvantage is the inability to separate debris and parasites, which results in a dense and filled smear of impurities^8^ ^,^ ^24^ ^,^ ^35^ ^,^ ^45^ ^,^ ^46^, even though decantation and resuspension are carried out in series and using fine mesh sieves^24^. The interference of these residues is related to adherence and overlapping of the parasitic structure and parasite-like artifacts and this condition reduces technical sensitivity^8^ ^,^ ^24^ ^,^ ^43^ ^,^ ^45^.

### The technique for larvae identification

The first technique developed for nematode larvae identification was described in 1917 by Baermann, who could isolate them from the soil by positive hydrotropism^36^. This technique was adapted for research on parasites in human feces by Moraes^37^ and was renamed as the Baermann-Moraes technique. Although Coutinho et al*.* made another modification, their study is rarely mentioned in the literature^87^ ^-^ ^89^. The Baermann-Moraes technique was incorporated into the laboratory routine at the Adolfo Lutz Institute by Rugai, Mattos, and Brisola when they verified the high incidence of *Strongyloides stercoralis* in the samples sent to that institution for research on intestinal parasites. However, due to the lack of materials described to perform the technique, the use of a conical bottom cup and pipettes was introduced^90^.

Other techniques for identifying larvae of intestinal parasites, such as that of Harada-Mori, modified by Sasa et al.^91^ and Hsieh^38^ ^,^ ^91^ ^,^ ^92^, promote the hatching of nematode eggs by inoculation onto specific media and filter paper arranged in Petri dishes^93^ or even charcoal^8^. The cultivation of larvae of *S. stercoralis* on agar plates was developed by Arakaki et al.^94^ and modified by Koga et al.^95^. Due to a higher sensitivity of these techniques for detection of larvae of *S. stercoralis*, more time and work is required for their employment. There is also limited exclusive detection of nematodes in larval forms and a need of fresh samples containing live larvae for their performance. Therefore, these techniques^82^ are generally not incorporated into the laboratory routine.

## ALTERNATIVE TECHNIQUES

The limitations of the techniques presented here are related to difficulties in harvesting and inadequate conservation of the biological sample^23^ ^,^ ^47^ ^,^ ^60^; concealment of parasitic structures in fecal smear debris^96^; damage to the structure of the evolutionary forms of the parasites by use of chemical reagents^66^ ^,^ ^85^; parasitic loss through numerous physicochemical stages^27^ ^,^ ^41^; restriction of the quantity of species recovered; inadequate knowledge; interpretation, and yield (fatigue) of the microscopist in extensive routines^50^ ^,^ ^96^ ^,^ ^97^. All these characteristics may interfere with the result and, consequently, with the sensitivity of the method. 

Due to these deficiencies, it was suggested by several researchers^98^ ^,^ ^99^ that the most efficient way to diagnose all species would be the simultaneous application of more than one technique^8^. However, this measure may make it challenging to perform the test per sample, due to the increase in test result time. An alternative is using commercial kits developed according to the same technical principles (Table 1). Despite the tendency to use kits routinely, it is still necessary to improve and develop innovative techniques and principles for the detection and quantification of intestinal parasites^8^.

**TABLE 1:** Commercial kits developed for the processing of human fecal samples.

Commercial kits	Recovery Principle	Manufacturer	Country	Ref.
Bioparaprep Mini^®^	Centrifugation-sedimentation	Leti Diagnostics	Spain	^98^
Coproplus^®^ (Coprotest)	Spontaneous sedimentation	NL Diagnóstica	Brazil	^55^ ^,^ ^56^
Copropack^®^	Centrifugation-sedimentation	Química Clínica Aplicada S.A	Spain	^99^
Midi Parasep^®^	Centrifugation-sedimentation	Apacor	England	^27^ ^,^ ^100^
Para-Pak^®^	Centrifugation-sedimentation	Meridian Bioscience, Inc.	USA	^101^ ^,^ ^102^
Paratest^®^	Spontaneous sedimentation	DK Diagnostics	Brazil	^43^
Para-Uni System^®^	Centrifugation-sedimentation	Biolife	Italy	-

To improve the diagnostic sensitivity of the parasitological examination of feces, Gomes et al*.* developed a qualitative parasitological technique called TF-Test or Three Fecal Test^45^. This technique consists of a) TF-Test kit (Biobrasil Ciência e Tecnologia) composed of three collection tubes containing 5 mL of buffered neutral formalin solution for fecal sample collection, and b) operational protocol for fecal sample processing based on the centrifugal-sedimentation principle with formalin-ethyl acetate. This protocol proposed a single laboratory processing of three fecal samples obtained on alternate days, which favored a more effective diagnosis^45^ ^,^ ^48^.

A study carried out in Brazil with 377 individuals showed greater positivity of the TF-Test technique in detecting protozoa and helminths when compared with the techniques of Kato-Katz, Hoffman, Pons and Janer, Willis and Baermann-Moraes^48^. Siqueira et al. evaluated the TF-Test technique for the detection of *S. mansoni* eggs, with results similar to those observed during the analysis of 12 slides using the Kato-Katz technique. The use of only 500 mg of feces in this study, an amount not standardized in the technique protocol, may, however, underestimate the performance of the technique^100^. A new study carried out by Nacife et al. showed less positivity for the TF-Test technique in the detection of *S. mansoni*, and higher positivity for other helminths and protozoa than the Kato-Katz technique. In this study, however, only one fecal sample from each individual was analyzed for logistical reasons, rather than three as in the protocol^101^.

 Carvalho et al*.* added a second centrifugation step to the technique to remove fecal impurities from the sediment; this favored the characterization of the parasitic form and reached a diagnostic sensitivity of up to 98%^46^ ^,^ ^102^. Further field studies with a more significant number of participants are needed to ensure the diagnostic sensitivity of this technique with more precision. To promote greater sensitivity in the identification of eggs of *S. mansoni*, a new technique called TF-Test Quantified was recently validated in the laboratory^103^. This technique establishes a serial collection of fecal material and is based on concentrating the parasite via the centrifugal-sedimentation principle and application of a conversion factor for eggs per gram of feces (EPG) determination.

The technique standardized by Cringoli, called FLOTAC, involved the use of centrifugal flotation with a technological device and operational protocol^104^. The procedure involves the dilution of 1 g of feces in a preservative solution, followed by filtration and addition of a solution for the flotation of parasitic structures using a specific apparatus developed for the technique. This technique evaluated several flotation solutions (FS) at variable densities and verified the recovery and quantification of several helminth species in humans and animals^41^ ^,^ ^105^. The FLOTAC technique was compared with conventional quantification techniques and was shown to be efficient with superior results for sensitivity^29^ ^,^ ^60^ ^,^ ^82^ ^,^ ^106^ ^,^ ^107^ but did not demonstrate the same performance in protozoan infections^71^ ^,^ ^83^ ^,^ ^108^. The Mini-FLOTAC technique was created to simplify the procedure in the laboratory, eliminating the need to use a centrifuge.

Another kit, the Helm-Teste kit, based on the Kato-Katz technique, proved to be feasible in routine applications and research due to simplicity and practicality^48^. It was presented in 2010 by the Oswaldo Cruz Foundation (FIOCRUZ), is marketed by Instituto de Tecnologia em Imunobiológicos (BIOMANGUINHOS), and currently recommended by the Ministry of Health of Brazil for diagnosis and control of *S. mansoni*. This commercial kit contains all the necessary material for slide preparation using the protocol of the Kato-Katz technique for quantitative detection of *S. mansoni* eggs by estimating the number of EPG.

Other studies have explored the magnetic attraction exerted on the surface of parasitic structures for recovery of eggs of *S. mansoni* and oocysts of *Cryptosporidium* spp^109^ ^-^ ^112^. Teixeira et al. described a new diagnostic test designated Helmitex based on the magnetic attraction exerted on the eggs membrane surface for recovery of *S. mansoni* with the use of magnetic microspheres^110^. This technique can achieve up to 100% sensitivity in samples with 1.3 EPG and therefore be used in regions where there is low infection intensity^111^ ^,^ ^113^ ^,^ ^114^.

 The perspectives for test examination are related to automation of the microscopic diagnosis, which may exclude the interpretation bias of the microscopist, assuring uniformity and quality control of the microscopic evaluation^97^. Yang et al. presented an algorithm capable of analyzing digital images of fecal samples preserved in formalin containing seven species of helminths, which reached 84% detection with digital processing and neural network classification of 82 images^115^. Similar results were obtained by Jiménez et al., who developed an algorithm capable of identifying seven species of helminths in images obtained from environmental samples with 85% sensitivity^116^. Other methods of improvement in the automated detection of parasites were proposed^117^ ^,^ ^118^; however, they involved the use of pre-selected images.

In 2013, Suzuki et al. presented a system composed of a parasitological protocol, a computer-controlled microscope with a digital camera, and computational techniques for the automated detection of 15 species of human parasites, including protozoa and helminths^119^. Fecal samples were collected in the field and processed using the TF-Test Modified parasitological technique^102^, and then slides are scanned and processed by the computer. Computational techniques perform three main steps: image segmentation, object representation, and object recognition. The first step locates and delineates candidate objects in the image, which may contain various parasite structures, such as helminth eggs and larvae, protozoan cysts and oocysts, and impurities with similar size, shape, color, and texture to those of parasites. The second step extracts characteristics of the candidate objects based on the color, shape, and texture, and the third step uses these characteristics to classify them as fecal debris or a species of parasite. This system reached 93% sensitivity and a kappa coefficient of 0.84^119^ ^,^ ^120^.

Another mathematical algorithm developed by Alva et al. was able to process images, extract characteristics, and classify images obtained from microscopy slides based on the characteristics of four helminth eggs^121^. Recently, Inácio et al. presented a new parasitological protocol for the detection of four genera of canine intestinal parasites and compared the manual reading of the slides with the automated system. The new parasitological protocol reached 80.88% of sensitivity in manual reading and 70.59% in the automated system^122^.

One of the difficulties for automation in parasitology is concentration of the parasite with the total elimination of debris without causing damage to the structural morphologies of the species^28^. Computational evaluation can accumulate accuracy, speed, and steady performance, provided that images with excellent quality and standardization are available to minimize the chances of error and confusion in computational analysis^97^ ^,^ ^117^ ^,^ ^120^. 

## CONCLUSION

Challenges associated with the techniques related to the parasitological examination of feces span over a century of scientific and technological development, with advances that enabled an increasingly precise diagnosis of intestinal parasitosis by microscopy. The technique to be used for diagnosis should be decided on the basis of the diagnostic purpose, available resources, and quantity required of examinations. Currently, the use of molecular methods with higher diagnostic precision, such as PCR, is recommended for morphologically indistinguishable species; however, its use also depends on the scope of research. In the laboratory, kits (centrifugation/flotation) have similar accuracy to conventional techniques and satisfy the diagnostic needs and operating cost. However, the limiting effects of the technical-operational, biological, and physicochemical factors inherent to the technical procedures used in fecal processing for diagnosis are still being investigated.
